# Genomic Characterization and Pathogenicity Island Analysis of 17 Mexican Isolates of *Corynebacterium pseudotuberculosis* biovar *ovis*

**DOI:** 10.3390/cimb48060598

**Published:** 2026-06-05

**Authors:** Mabel Gethsemani Jaimes-Gonzalez, Roberto Montes-de-Oca-Jimenez, Martha Elba Ruiz-Riva-Palacio, Gabriel Arteaga-Troncoso, Jorge Pablo Acosta-Dibarrat, Pilar Eliana Rivadeneira-Barreiro, Pablo Cleomenes Zambrano-Rodriguez, Dan Israel Zavala-Vargas, Siomar de Castro Soares, Victor Augusto Sallum Ceballos, Pedro Sanchez-Aparicio, Vasco Ariston de Carvalho Azevedo

**Affiliations:** 1 Research and Advanced Studies in Animal Health Center, Faculty of Veterinary Medicine and Zootechnics, Autonomy University of the State of Mexico, Km 15.5 Toluca Pan-American Highway Atlacomulco, Toluca C.P. 50200, State of Mexico, Mexico; magejago28@gmail.com (M.G.J.-G.); jpacostad@uaemex.mx (J.P.A.-D.); danisraelz@gmail.com (D.I.Z.-V.); psanchezap@uaemex.mx (P.S.-A.); 2 Sor Juana Ines de la Cruz School, Autonomy University of the State of Mexico, Amecameca, Amecameca de Juarez C.P. 56900, State of Mexico, Mexico; meruizr@uaemex.mx; 3 Department of Biology and Cell Development, National Institute of Perinatology, Chapultepec Hills Sec. IV, Miguel Hidalgo, Mexico City C.P. 11000, Mexico; drgarteagat@yahoo.com.mx; 4 Department of Veterinary Medicine, Faculty of Veterinary Sciences, Technical University of Manabi, Urbina Avenue, Portoviejo C.P. 130105, Ecuador; pilar.rb26@hotmail.com (P.E.R.-B.); drpablozambrano@gmail.com (P.C.Z.-R.); 5 Chimalhuacan General Hospital, IMSS-Wellness, Av. Del Peñon Mza. 400 Lt. 1 Canteros Neighborhood, Chimalhuacan C.P. 56330, State of Mexico, Mexico; 6 Department of Microbiology, Immunology and Parasitology, Federal University of Triangulo Mineiro, Av. Frei Paulino, 30-Nossa Sra. da Abadia, Uberaba C.P. 38025-180, Minas Gerais, Brazil; siomars@gmail.com (S.d.C.S.); victoraugusto_sc@hotmail.com (V.A.S.C.); 7 Department of Genetics, Ecology, and Evolution, Federal University of Minas Gerais, Av. Antonio Carlos, 6627, Pampulha, Belo Horizonte C.P. 31270, Minas Gerais, Brazil; vascoariston@gmail.com

**Keywords:** *Corynebacterium pseudotuberculosis*, pathogenicity islands, virulence factors, caseous lymphadenitis

## Abstract

Pathogenicity islands (PAIs) are regions of bacterial genomes that harbor genes encoding virulence factors. Identifying molecules that enhance pathogenicity is crucial for understanding the mechanisms pathogens employ to cause disease and their evolution. *Corynebacterium pseudotuberculosis* (*C. pseudotuberculosis*) is a pathogenic microorganism that causes caseous lymphadenitis (CLA) in sheep and goats. Despite its prevalence in Mexico, its genetic material has not been analyzed for virulence factors acquired through horizontal gene transfer. Therefore, the aim of this study was to characterize the complete genomes of Mexican *C. pseudotuberculosis* strains and identify virulence-related genes harbored with PAIs. Seventeen strains of *C.pseudotuberculosis* biovar *ovis* isolated from Mexico were whole-genome sequenced using illumina technology, assembled de novo with SPAdes, and annotated using Prokka. PAIs were predicted with GIPSy based on genomic signatures associated with horizontal gene transfer, including G + C deviation, codon usage, virulence factors, transposases, and tRNA-flanking regions. Positive selection was assessed using POTION v1.2 by identifying orthologous groups enriched in non-synonymous substitutions. This represents the first comprehensive PAI analysis of Mexican *C. pseudotuberculosis* strains, identifying 14 putative pathogenicity islands harboring 51 virulence-associated genes. Additionally, positive selection analysis identified five coding sequences, including *radA* and *rpiB*, that are undergoing adaptive evolutionary changes. These findings elucidate the pathogenic mechanisms and genomic plasticity of Mexican *C. pseudotuberculosis* strains. They also highlight novel genetic targets for vaccine and therapeutic development against CLA.

## 1. Introduction

*Corynebacterium pseudotuberculosis* is a facultative intracellular Gram-positive bacterium that causes caseous lymphadenitis (CLA), a chronic infectious disease characterized by abscess formation in superficial and internal lymph nodes of small ruminants [1].

*C. pseudotuberculosis* is classified into two biovars based on nitrate reductase activity: biovar *ovis* (nitrate-negative), which predominantly affects sheep and goats and causes caseous lymphadenitis (CLA), and biovar *equi* (nitrate-positive), which primarily infects horses and causes ulcerative lymphangitis, pectoral abscesses, and internal organ infections [2]. Mexico harbors a significantly small ruminant population (approximately 8.8 million sheep and 8.7 million goats), with CLA representing a major cause of economic losses due to carcass condemnation, reduced wool and milk production, and increased culling rates [2,3]. Despite this economic impact, the genomic characterization of Mexican *C. pseudotuberculosis* strains, particularly their virulence gene repertoire acquired through horizontal gene transfer, has remained unexplored. The implementation of massive sequencing technologies has enabled the characterization of the complete gene repertoire and virulence-associated determinants of *C. pseudotuberculosis*. To date, more than 238 genomes are publicly available in the NCBI database, including the reference strain Cp 1002, widely used in comparative genomic analyses. Previous studies have reported the genomes of six Mexican strains belonging to *C. pseudotuberculosis* biovar *ovis* and *equi* [4]. However, genomic studies focused on Mexican isolates remain limited, despite the endemic occurrence of CLA in several sheep- and goat-producing regions of the country. Moreover, evidence of horizontal gene transfer in *C. pseudotuberculosis* suggests that the acquisition of adaptive and virulence-related traits contributes to the evolution and pathogenicity of this species [5].

Pathogenicity islands (PAIs) are genomic regions acquired through horizontal gene transfer that harbor virulence-associated genes involved in host adaptation, survival, and infection. These regions are commonly identified using genomic signatures such as deviations in G + C content, codon usage, transposases, virulence genes, and flanking tRNA sequences. Previous studies identified seven PAIs in *C. pseudotuberculosis*, including regions containing the *pld* gene, an important virulence determinant and vaccine target [6,7]. Subsequent pan-genomic analyses revealed additional putative PAIs and candidate virulence genes, highlighting the genomic plasticity of the species [8].

In Mexico, CLA prevalence has been documented, and molecular diagnostic approaches, including techniques such as multiplex PCR, have been implemented for pathogen identification [9]. Nevertheless, comprehensive genomic characterization, phylogenetic relationships, and studies focused on virulence determinants and adaptive evolution in Mexican isolates are still scarce. Understanding the genetic basis of pathogenicity is essential for improving epidemiological surveillance [10,11], identifying vaccine candidates by selecting antigens for immunization [12,13], and monitoring strains dissemination, and evaluating the persistence of virulence-associated determinants linked to the host adaptation and environmental survival [14].

Despite the increasing availability of genomic data, no studies have systematically identified PAIs and positively selected genes in Mexican *C. pseudotuberculosis* isolates. The aims of this study were to: (i) sequence and assemble the complete genomes of 17 *C. pseudotuberculosis* biovar *ovis* isolates from Mexico; (ii) perform comparative genomic analysis with previously reported strains to determine phylogenetic relationships; (iii) identify and characterize putative pathogenicity islands harboring virulence factors acquired through horizontal gene transfer; and (iv) detect coding sequences under positive selection that may represent novel therapeutic or vaccine targets to identify adaptive evolutionary changes associated with pathogenicity and host adaptation of this microorganism. To date, no studies have systematically identified PAIs or positively selected genes in Mexican *C. pseudotuberculosis* isolates.

## 2. Materials and Methods

### 2.1. Study Methodology

**Figure 1:**
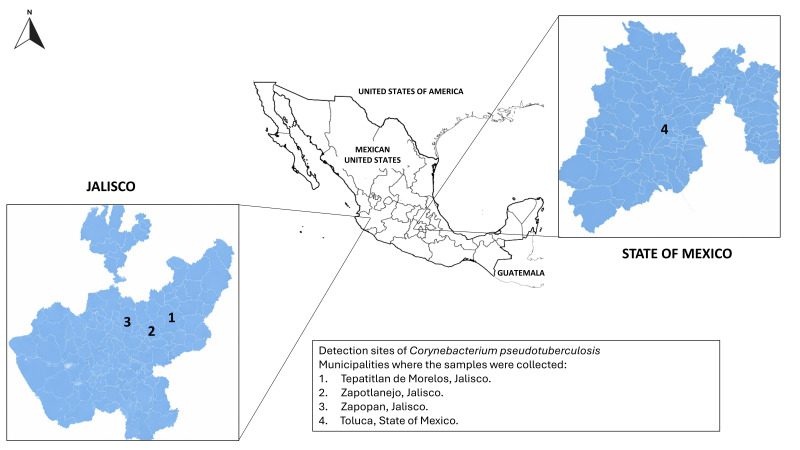
Geographic location map of the states where *Corynebacterium pseudotuberculosis* biovar *ovis* samples were collected. Detection sites of *Corynebacterium pseudotuberculosis*. Municipalities where the samples were collected: 1. Tepatitlán de Morelos, Jalisco; 2. Zapotlanejo, Jalisco; 3. Zapopan, Jalisco; and 4. Toluca, State of Mexico.

We analyzed data collected from a previous field study of 17 strains of *C. pseudotuberculosis* obtained from abscessed lesions of small ruminants [9]. The animals are located in a sheep-farming region of the Mexican highlands in the main municipalities of the state of Jalisco, Mexico. The livestock region includes 92,297 sheep and goats in the municipality of Tepatitlan, 174,964 in Zapopan, and 3628 in Zapotlanejo (Figure 1) [3].

These municipalities are located near the city of Guadalajara, where the first strains of *C. pseudotuberculosis* were detected. At the time, to reduce the risk of injury, spread, and death of the animals, as well as to ensure the safety of owners and researchers, we followed the guidelines, standards, and recommendations for good practices issued by Teagasc [15].

### 2.2. Ethical Implications

This study was conducted using archived bacterial isolates previously obtained from a field study [9] and stored in the Center for Research and Advanced Studies in Animal Health (CIESA) of the Faculty of Veterinary Medicine and Animal Science of the Autonomous University of the State of Mexico, Toluca, State of Mexico). No additional animal manipulation or experimentation was performed for this work. The study protocol was registered under number MACARN-1423 and was approved by the Institutional Ethics Committee of the Faculty of Veterinary Medicine and Animal Science of the Autonomous University of the State of Mexico.

### 2.3. Bacteriological and Molecular Identification of Corynebacterium pseudotuberculosis biovar ovis

The samples used in this study are stored in the laboratory of the UAEM Research and Advanced Studies in Animal Health Center (CIESA), Toluca, Mexico. *C. pseudotuberculosis* was identified by plating on blood agar (40 g/L-25 mL of sheep blood) to recognize and select colonies with characteristics indicative of *C. pseudotuberculosis*. Incubated at 37 °C for 24–48 h under anaerobic conditions. Pure colonies were then identified and cultured in 37 g/L Brain Heart Infusion (BHI) liquid medium through incubation for 48 to 72 h at 37 °C. Genetic materials were extracted following the instructions provided in the commercial kit FAST ID Genomic DNA Extraction (FANSA, Thermo Fisher, Carlsbad, CA, USA). A NanoDrop UV spectrophotometer and Nucleic Acid software (version 2.1) were used to evaluate DNA quality. The DNA concentration values obtained were recorded in ng/μL. To estimate DNA purity, absorbance at 260/280 nm was considered, with a ratio of 1.8 being accepted as pure DNA.

Molecular identification of *C. pseudotuberculosis ovis* isolates was performed using multiplex PCR amplification of fragments of four specific genes: *16s rRNA*, *rpoB*, *pld*, and *narG.* PCR was performed according to the protocol established by Almeida et al. (2017) [16], in a final volume of 50 μL containing 12.5 μL 2X Quiagen Multiplex PCR Master Mix, 1 μL for each primer (100 pmol/μL), and 2 μL of DNA (20 ng/μL). The thermocycler (Thermo Fisher, Vermon Hills, IL, USA) was used under the following conditions: initial denaturation at 95 °C for 4 min, 30 cycles of 95 °C for 30 s, 58 °C for 30 s, and 72 °C for 1.5 min, and a final extension at 68 °C for 7 min. The negative control consisted of a reaction with all components and nuclease-free water instead of the DNA sample. The products were visualized by electrophoresis on 1.5% agarose gels. The gels were subjected to 110 V for 45 min in a power supply (Hoefer Inc., Holliston, MA, USA) for the separation of DNA molecules. The gels were stained with ethidium bromide for DNA visualization using UV light in a transilluminator (Mini Bis Pro, DNR Bio-imagen System; Health Tech, and Life Sciences, Netanya, Israel). Molecular weight markers of 100 bp Plus DNA Ladder™ (Invitrogen, Carlsbad, CA, USA) and 1 Kb O’GeneRuler (Thermo Fisher, Carlsbad, CA, USA) were used.

### 2.4. Whole-Genome Sequencing

All genomes were sequenced by Illumina MiSeq (Library Preparation Kit: Nextera XT Library). The products obtained from the post-sequencing kit (Read size: 2 × 250 bp) were processed using SPAdes v3.15.3 software [17] following the de novo assembly methodology (Supplementary Materials: Metrics of the seventeen sequenced Mexican isolates). The quality of the assembly was subsequently assessed using FastQC v0.11.9 (quality assessment tool) and Quast v5.3.0 (assembly quality). We performed automatic genome annotation using Prokka software (version 1.14.6), a tool that identifies gene coordinates and assigns putative gene products [18].

### 2.5. Genomic Analysis of Corynebacterium pseudotuberculosis biovar ovis

The whole-genome sequences of the seventeen isolates of Mexican origin were aligned with 18 other genomes extracted from GenBank (Table S1) using the Gegenees program (version 2.1). Using the generated alignments, a heat map was constructed based on the percentage of similarity between the sequences. Finally, to evaluate the phylogenetic relationships of the Mexican isolates of *C. pseudotuberculosis*, a phylogenetic tree was constructed using the SplitsTree application (version 4.12.6), using the nexus file generated by Gegenees after alignment.

The positive selection analysis was performed using the POsitive selecTION pipeline (POTION) v1.2 in site-model mode (https://github.com/g1o/POTION (accessed on 10 July 2024 )). The orthogroup file generated by OrthoFinder was converted into an OrthoMCL-/compatible format and used as the homology input file for POTION. The analysis followed the default POTION configuration. POTION applied sequential quality-control filters to the coding sequences and homologous groups, including validation of start and stop codons, verification that sequence lengths were multiples of three, removal of sequences containing non-standard nucleotides, exclusion of sequences shorter than 100 bp, and filtering based on relative sequence length. The relative length thresholds were defined as 0.5 and 2.0 times the mean sequence length of each group. Homologous groups were retained only when they contained at least three genes and three genomes after filtering. Positive selection was inferred using codon substitution models implemented in PAML/codeml. The nested site-model comparisons M1a/M2a and M7/M8 were tested. Coding sequences were considered candidates under positive selection when the likelihood ratio test showed a *p*-value < 0.05 and a multiple-testing-corrected q-value < 0.05. Positively selected sites were interpreted as codons with dN/dS > 1 within the class of sites under positive selection, and not as evidence that the entire gene had a global Ka/Ks ratio > 1.

Whole-genome multilocus sequence typing (wgMLST) was performed using the cano-wgMLST pipeline [19]. Genome assemblies were annotated with Prokka [18], integrated into the pipeline, and the predicted coding sequences were used to construct a pangenomic allele database (PGAdb). The PGAdb generated in this analysis comprised 9310 loci. The dendrogram presented was generated using core wgMLST, corresponding to 79 loci retained in the core.scheme file. PGAdb construction was performed using a minimum BLASTP identity threshold of 95%. Subsequently, the allelic profile was generated by searching the allele database against each genome assembly using BLASTN, with a minimum aligned identity of 90% and a minimum alignment coverage of 0.9. Allele assignment required an exact match, defined as 100% identity, full-length alignment, absence of mismatches, and absence of gaps. The resulting allelic distance matrix was used to infer the wgMLST dendrogram using 100 bootstrap resamplings of the allelic profiles. The final tree was exported in Newick format and visualized in the Interactive Tree Of Life (iTOL) v6 web platform [20].

### 2.6. Identification of Pathogenicity Islands

We used the GIPSy software (version 1.1.3) to identify the coordinates of pathogenicity islands. GIPSy works by searching for characteristics linked to horizontal gene transfer: deviation from the genomic signature: G + C content (calculate G + C deviation on query genome using 1.5 as threshold; calculate G + C deviations on subject genome using 1.5 as threshold) and codon usage (define sensitivity to calculate codon usage deviation on query genome using 0.95 as threshold; define sensitivity to calculate codon usage deviation on subject genome using 0.95 as threshold); presence of transposase genes (search for transposase genes on query genome using 0.0001 as threshold); virulence factors (pathogenicity islands using 0.000001 as threshold); flanking tRNA genes (Search for tRNA genes in the query genome using 0.0001 as threshold); and absence in other organisms of the same genus or closely related species. This methodology highlights the horizontal transfer processes that occur in pathogenic microorganisms, improving our understanding of bacterial genome plasticity and adaptation to new hosts. Specifically for the identification of virulence factors, protein similarity searches are performed using the blastp algorithm in the mVIRdb virulence factor database. mVIRdb contains protein sequences from ten different databases: Tox-Prot, SCORPION, PRINTS virulence factors, VFDB, TVFac, Islander, ARGO, CONUS, and a subset of VIDA [21]. The GIPSy results generate a coordinate format that must then be analyzed using Artemis 18.2.0 software to identify gene products within the regions.

Additionally, we predicted the Pathogenicity Islands of *C. pseudotuberculosis* strains 1002 and CIP 52.97 (biovars *ovis* and *equi*, respectively), *Corynebacterium ulcerans* BR-AD22 and *Corynebacterium diphtheriae* NCTC 13129 all using *Corynebacterium glutamicum* ATCC 13032 as non-pathogenic reference in GIPSy with standard parameters. Afterwards, we used in-house scripts to fragment the PAIs from each strain in sequences of 500 nucleotides with an overlap of 100 nucleotides, to obtain the amino acid sequences from the six open reading frames, to align the sequences with the software diamond, to retrieve only the best alignment for each fragment and to create a distance matrix from the percentage of shared fragments. Finally, the distance matrix was used to plot a heatmap and a Neighbor Joining phylogenetic tree based on all strains.

## 3. Results

### 3.1. Identification of Corynebacterium pseudotuberculosis Isolates of Mexican Origin

**Figure 2:**
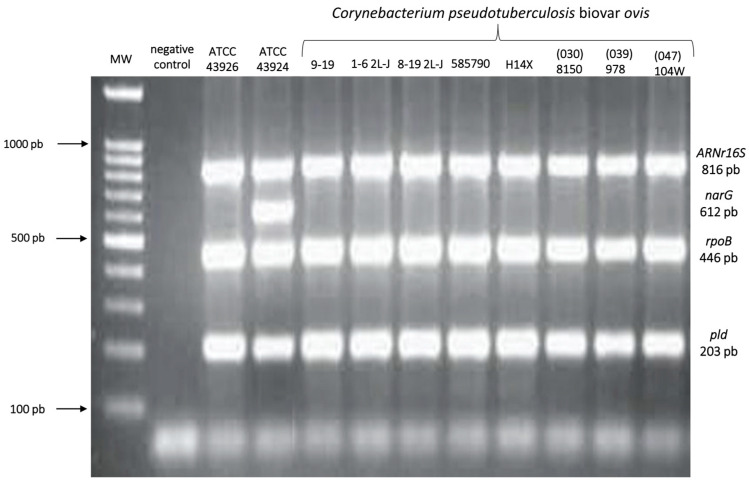
Quadruplex PCR for biovar differentiation of *C. pseudotuberculosis isolates.* Lanes: MW = molecular weight marker (catalog number of the 250 µL/G2101 molecular weight marker); NC = negative control; *Ovis*+ = positive control ATCC 43926 (biovar *ovis*); *Equi*+ = positive control ATCC 43924 (biovar equi); lanes 5–12 = representative Mexican isolates (9-19, 1-6 2L-J, 8-19 2L-J, 585790, H14X, (030) 8150, (039) 978, (047) 104W).

Seventeen strains of *C. pseudotuberculosis* were isolated from abscess lesions obtained from fourteen sheep, two goats, and one bovine. The strains obtained from sheep and goats originated in the State of Jalisco, Mexico and the bovine strain originated in the State of Mexico, Mexico (Table 1). All samples were confirmed by quadruplex PCR and corresponded to biovar *ovis* (Figure 2). Positive control ATCC 43926 (biovar *ovis*) and positive control ATCC 43924 (biovar *equi*) were used. The nomenclature used for the isolates was in accordance with the ear tag number of each of the animals from which the isolate was obtained. All animals have an individual record in the sheep or goat production units.

### 3.2. Sequencing and Assembly of the Complete Genome

The sequencing and genomic assembly resulted in the draft genome sequence, in FASTA format, of the seventeen isolates of Mexican origin, all corresponding to the microorganism *C. pseudotuberculosis*, with a genome size ranging from 2,331,531 bp (033B) 635816) for the smallest genome to 2,337,637 (585790) for the largest genome. All had a G + C content of 52.18% and a total number of genes between 2129 and 2138 (Table 2).

All sequenced and assembled genomes were analyzed with the Quast program, obtaining the following values: N50 $\bar{x}$ = 2,334,730 (mean) and L50 = 1.

### 3.3. Comparative Genomic Analysis of Corynebacterium pseudotuberculosis Isolates of Mexican Origin

**Figure 3:**
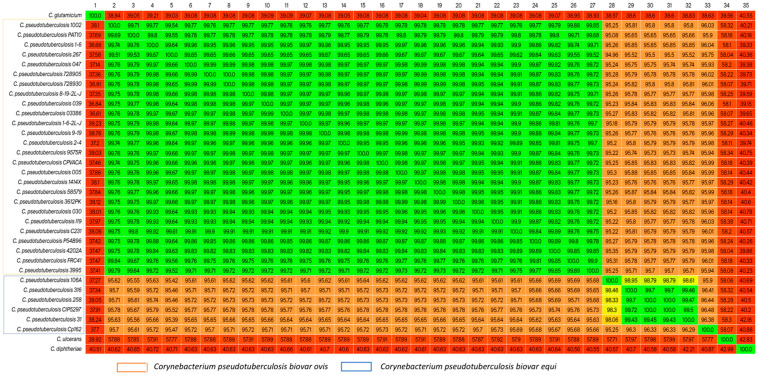
Heatmap of *Corynebacterium pseudotuberculosis* genomes of Mexican origin. Sequenced genomes of Mexican origin and other strains from the GenBank database were used to generate a heat map based on the percentage similarity of their sequences. The numbers from 1 to 35 represent the strains shown on the left side of the graph, in the same order. The similarity percentages are displayed graphically, from green (high similarity) to red (low similarity). Genomes of Mexican origin formed a high-percentage-of-similarity group along with other genomes of the biovar *ovis*. Meanwhile, genomes of the biovar *equi* also formed a common group due to a higher percentage of intra-biovar similarity.

**Figure 4:**
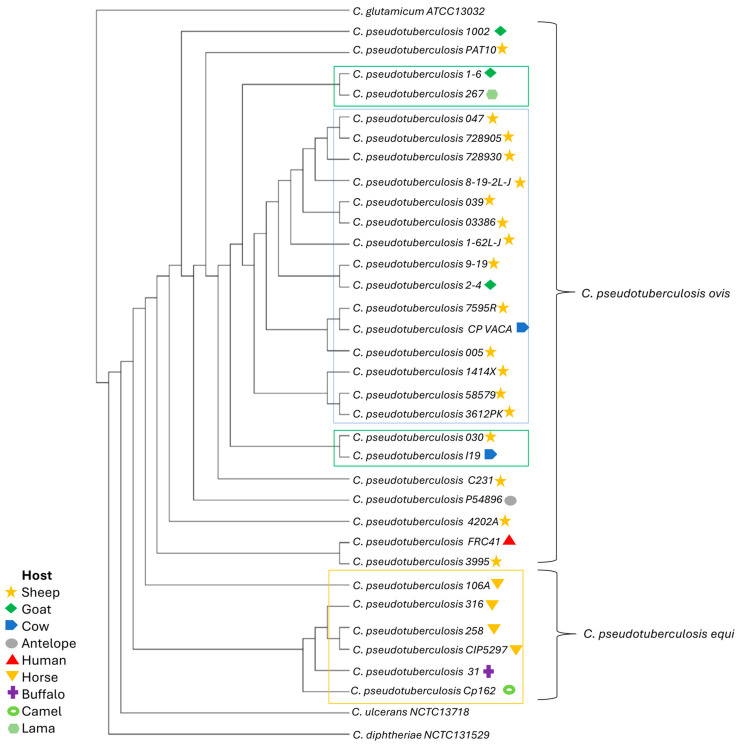
Whole-genome phylogenetic tree of Mexican isolates of *Corynebacterium pseudotuberculosis* biovar *ovis*. The phylogenetic relationships of new isolates of *Corynebacterium pseudotuberculosis* of Mexican origin and other pathogenic strains of *C. pseudotuberculosis*, *C. diphtheriae* and *C. ulcerans* were analyzed. We also included the genome of a non-pathogenic species of the genus *Corynebacterium (C. glutamicum).* All Mexican isolates were closely related, except for isolates 1-6 and 030, which formed clades with strains 267 and i19, respectively. The non-pathogenic strain *C. glutamicum* was shown to be distant from the pathogenic strains of the genus *Corynebacterium*, generating a distant branch. Blue box: *C. pseudotuberculosis* biovar *ovis* of Mexican origin; Yellow box: *C. pseudotuberculosis* biovar *equi.* Green box: *C. pseudotuberculosis* biovar *ovis* of Mexican origin that joins in a clade together with strains of different geographical origin.

The complete sequences were analyzed using Gegenees (version 2.1), and an alignment file was generated, which was then plotted on a heat map based on similarity percentages (Figure 3). Strains of Mexican origin formed a single group with other strains of the *ovis* biovar, showing high similarity of 99.6–100%. The *equi* biovar strains also formed a group, with similarity percentages ranging from 99.4% to 100%, indicating greater intra- than inter-biovar similarity. We used SplitsTree (version 4.12.6) to evaluate phylogenetic relationships (Figure 4). The new strains of *Corynebacterium pseudotuberculosis* of Mexican origin were closely related and clustered. Strains 1–6 and 030 formed clades with strains 267 (Lama/USA) and i19 (Vaca/Israel), respectively. The non-pathogenic strain *C. glutamicum* was distantly related to the pathogenic strains of the genus *Corynebacterium*, forming a separate branch.

Positive selection analysis identified five coding sequences (CDS) with evidence of adaptive evolution according to the POTION site-model criteria. These CDS included genes annotated as *radA*, encoding a DNA repair and recombination-associated protein, and *rpiB*, encoding ribose-5-phosphate isomerase B, as well as three hypothetical proteins. None of these CDS were located within the predicted pathogenicity island coordinates. These genes were interpreted as candidate loci under positive selection and not as experimentally validated virulence determinants.

**Figure 5:**
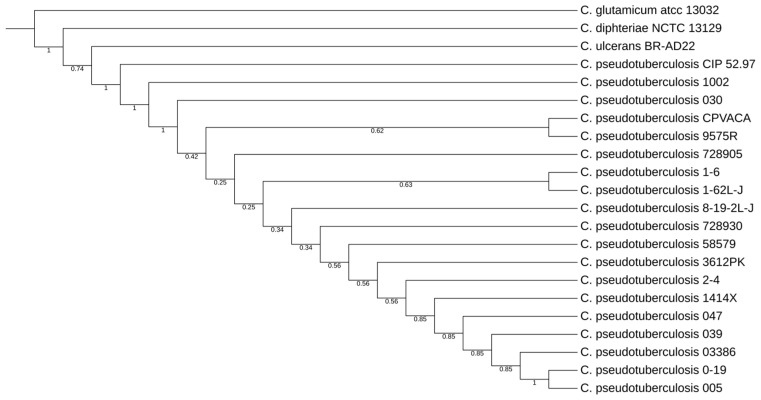
Phylogenetic tree of *Corynebacterium pseudotuberculosis* biovar *ovis* of Mexican origin using whole genome multilocus sequence typing (wgMLST). The sequences of Mexican-origin *C. pseudotuberculosis* biovar *ovis* strains were used in conjunction with other strains of the genus *Corynebacterium* (*C. pseudotuberculosis 1002 biovar ovis*, *C. pseudotuberculosis CIP52.97 biovar equi*, *C. ulcerans*, *C. diphtheriae*, *C. glutamicum*) to analyze their phylogenetic relationships using whole-genome multilocus sequence typing (wgMLST). This revealed the close relationship between the Mexican strains and strain 1002, as well as the distance between *C. pseudotuberculosis* and *C. glutamicum*.

In addition to a detailed analysis of the taxonomic relationships of Mexican isolates of *Corynebacterium pseudotuberculosis* biovar *ovis*, allelic profiling was performed using whole-genome multilocus sequence typing (wgMLST). The final wgMLST dendrogram was generated from 79 core loci retained from a PGAdb of 9310 loci, using pairwise allelic differences among genomes (Figure 5). The resulting UPGMA dendrogram showed that the Mexican *C. pseudotuberculosis* biovar *ovis* isolates were highly similar at the allelic-profile level. Specifically, strains CPVACA and 9575R grouped in the same clade, as did the group composed of 1-6, 1-6 2L-J, 9-19, and 005. As expected, strain 1002, isolated from a goat in Brazil and belonging to biovar *ovis*, was closest to the Mexican isolates. In contrast, strain CIP 52.97, isolated from a horse in Kenya and belonging to biovar equi, was more distant. The *C. ulcerans*, *C. diphtheriae*, and *C. glutamicum* reference genomes were positioned more distantly, consistent with their taxonomic separation from *C. pseudotuberculosis*.

### 3.4. Prediction of Pathogenicity Islands in Mexican Isolates of Corynebacterium pseudotuberculosis

The 17 sequenced genomes were analyzed using GIPSY software (version 1.1.3.). The results obtained reflect the coordinates of the putative pathogenicity islands. Subsequently, the genes located within these coordinates in each PiCp (Putative Pathogenicity Island of *Corynebacterium pseudotuberculosis*) were identified. The identified genes and the proteins they encode are described in Table 3.

### 3.5. Sequence Similarity Analysis of Regions Identified as Pathogenicity Islands Between Mexican Isolates of Corynebacterium Pseudotuberculosis and Other Strains of the Genus Corynebacterium

**Figure 6:**
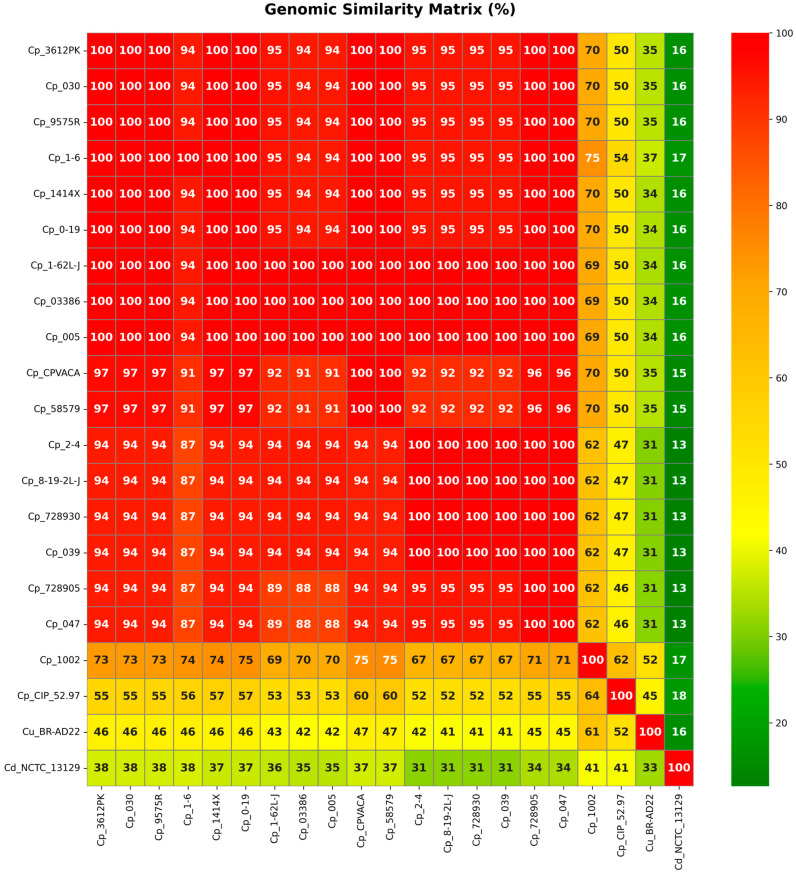
Heat map of pathogenicity island sequences from Mexican-origin *Corynebacterium pseudotuberculosis* strains and other strains of the genus *Corynebacterium*. Cp_3612PK to CP_1002: *Corynebacterium pseudotuberculosis biovar ovis*. Cp_CIP_52.97: *Corynebacterium pseudotuberculosis biovar equi.* Cu_BR-AD22: *Corynebacterium ulcerans.* Cd_NCTC_13129: *Corynebacterium diphtheriae*.

**Figure 7:**
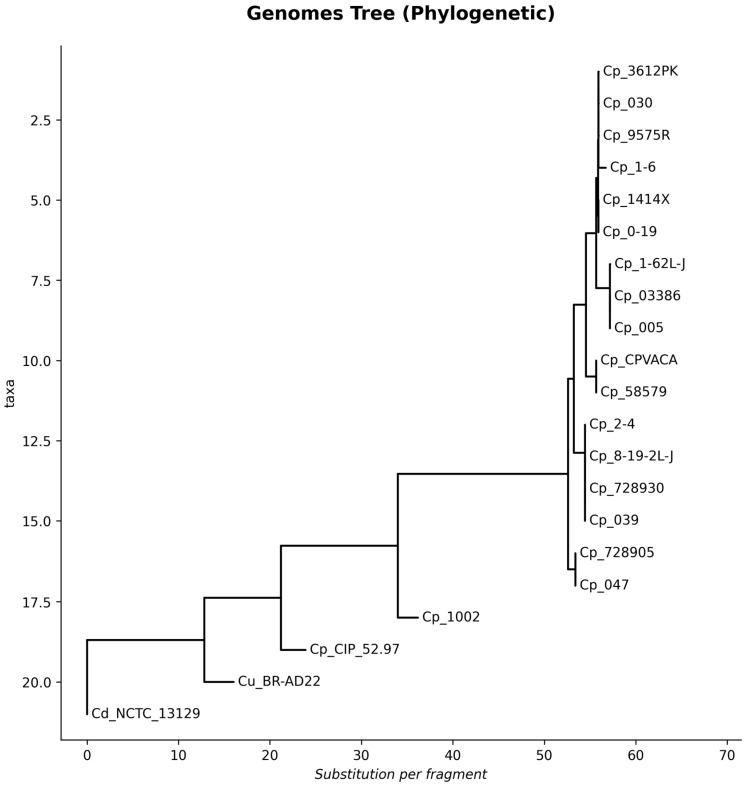
Phylogenetic tree of pathogenicity island sequences from Mexican strains of *Corynebacterium pseudotuberculosis.* Cp_3612PK to CP_1002: *Corynebacterium pseudotuberculosis biovar ovis*. Cp_CIP_52.97: *Corynebacterium pseudotuberculosis biovar equi.* Cu_BR-AD22: *Corynebacterium ulcerans.* Cd_NCTC_13129: *Corynebacterium diphtheriae*.

The distance matrix generated from the percentage of shared fragments is used to plot a heat map (Figure 6) and a neighbor-joining phylogenetic tree (Figure 7) based on all strains. Similarity analysis of sequences between pathogenicity island regions of Mexican *Corynebacterium pseudotuberculosis* strains and other strains of the genus *Corynebacterium* showed high percentages of similarity among the Mexican-origin sequences, demonstrating the conservation of regions. We identified a decrease in similarity values between sequences of the Mexican strains and the *C. pseudotuberculosis* strain 1002. The similarity values between the Mexican isolates and the *C. ulcerans* and *C. diphtheriae* strains were significantly lower. The phylogenetic tree generated from the alignment of sequences of regions identified as pathogenicity islands shows close proximity among the sequences of *C. pseudotuberculosis* strains of Mexican origin. The PiCp sequences of strains CPVACA and 58579 clustered in the same clade, as did the groups formed by strains 728905 and 047/2-4,8-19-2L-J, 728930, and 039. Strain 1002 is found in close proximity to the Mexican strains, followed by strain CIP52. The PiCp sequences of the species *C. diphtheriae* are more distant.

## 4. Discussion

### 4.1. Comparative Genomic Analysis of Corynebacterium pseudotuberculosis biovar ovis of Mexican Origin

#### 4.1.1. Phylogenetic Relationships of *Corynebacterium pseudotuberculosis* biovar *ovis* of Mexican Origin

The phylogenetic relationships of *C. pseudotuberculosis* have been documented since the introduction of sequencing technologies, thanks to the open access database generated from sequenced genome information. The results of the phylogenetic analysis performed in our study show that the biovar *ovis* strains formed common clades and were separated from the group formed by the biovar *equi* strains. *C. pseudotuberculosis* was found to be closer to *C. ulcerans* and *C. diphtheriae*, pathogenic species of the same genus. These results coincide with the data generated by the phylogenetic evaluation of Soares et al. (2013) [6], who considered multiple species of the genus *Corynebacterium*, highlighting greater proximity to *C. ulcerans* and *C. diphtheriae*, in contrast the non-pathogenic species *C. glutamicum* established a distant genomic branch, highlighting the evolutionary divergence between pathogenic species adapted to the environment and those adapted to the host [1]. In addition, alignments were generated that confirm the formation of two groups, comprising the strains of biovar *ovis* and biovar *equi*. A higher percentage of similarity was calculated between the biovar *ovis* strains (99%) in contrast to biovar *equi* (95%); these results coincide with those obtained in the alignments of the Mexican isolates, which show 99% intra-biovar similarity. These data confirm the clonal behavior previously proposed by Soares et al. (2013) [6], who postulate greater plasticity in the *C. diphtheriae* genome. Parise et al. (2018) [4] considered the members of the CMNR group (*Corynebacterium*, *Mycobacterium*, *Rhodococcus*, and *Norcardia*), closely related species that showed clades distinct from *Corynebacterium* strains, in the generation of the phylogenetic tree [4]. This conservation could reflect selective pressures associated with survival in small ruminants and adaptation to chronic abscess formation. Similar genomic stability has been reported in intracellular pathogens that persist in host tissues for extended periods, where lower genomic variability contributes to the maintenance of virulence-associated traits [7]. Comparison with globally characterized strains reveals both conservation and divergence. The Brazilian reference strain 1002 (biovar *ovis*) shares the highest PAI sequence similarity with the Mexican isolates, consistent with the clonal nature of biovar *ovis*. Strain 267, isolated from a llama in the United States, and strain i19, isolated from a cow in Israel, clustered with Mexican isolates 1-6 and 030, respectively, suggesting cross-host and cross-geographic conservation of core virulence gene repertoires. However, the absence of *pilus* genes (*spaA* and *spaD* clusters) in the Mexican genomes, which were identified in the pan-genomic study by Soares et al. (2013) [6], indicates lineage-specific gene loss that may reflect adaptation to local host populations or ecological conditions. The *equi* biovar strains (e.g., CIP 52.97) showed lower PAI sequence similarity, consistent with the known genomic divergence between biovars.

#### 4.1.2. Phylogenetic Relationships of *Corynebacterium pseudotuberculosis* biovar *ovis* of Mexican Origin: Whole Genome Multilocus Sequence Typing (wgMLST) Method

WgMLST analysis further confirmed the close genetic relationship among the Mexican isolates, grouping several strains within the same clones despite originating from different sites in municipalities of Jalisco, Mexico, and from their hosts. These observations support the hypothesis that the spread of *C. pseudotuberculosis* in Mexican livestock systems could involve the regional circulation of highly related clones. The movement of animals between production systems, shared grazing areas, and the environmental persistence of the microorganism could contribute to the maintenance of genetically similar lineages in endemic regions. Similar approaches based on wgMLST have been applied with great success to high-resolution epidemiological analyses of some bacterial pathogens [22]. The results showed the close relationship between strains of Mexican origin, grouping some into the same clade of origin (CPVACA, 9575R/1-6 and 1-62l-J).

#### 4.1.3. Positive Selection Analysis of *Corynebacterium pseudotuberculosis* biovar *ovis* of Mexican Origin

The *radA* gene encodes a DNA repair protein that facilitates recombinational repair of stalled replication forks. Positive selection in *radA* may reflect adaptation to oxidative stress encountered within host macrophages, where DNA damage is prevalent. In *E. coli*, *RadA* deletion increases UV sensitivity [23], suggesting that enhanced *RadA* function could confer a survival advantage during intracellular persistence. The *rpiB* gene encodes ribose-5-phosphate isomerase B, an enzyme in the pentose phosphate pathway that generates NADPH and ribose-5-phosphate for nucleotide biosynthesis. Positive selection in *rpiB* may indicate metabolic adaptation to the nutrient-limited intracellular environment. In Leishmania infantum, *RpiB* was validated as a pharmacological target essential for parasite survival [24]. The adaptive evolution of these two genes suggests selective pressure related to intracellular survival and metabolic fitness, although their direct contribution to virulence in *C. pseudotuberculosis* requires experimental confirmation.

### 4.2. Putative Pathogenicity Islands of Corynebacterium pseudotuberculosis biovar ovis of Mexican Origin

Genomic sequencing increases the possibility of pinpointing mechanisms of pathogenicity and lifestyle of pathogens [7]. The identification of PAIs has revealed that the genes they harbor play a fundamental role in various phases of infection, intracellular survival, magnesium and iron absorption, resistance to multiple antimicrobials, and the development of systemic infections by the microorganism [25]. In the present study, we identified the complete genomes of *C. pseudotuberculosis* of Mexican origin that harbor genes involved in pathogenicity for the first time. The GIPSY package was used to identify fourteen regions with characteristics linked to horizontal gene transfer. Few studies have reported the identification of these regions in *C. pseudotuberculosis.* Ruiz et al. (2011) [7]. first reported seven pathogenicity islands (PAIs) in two *C. pseudotuberculosis* strains (Cp1002/goat native to Brazil and CpC231/sheep native to Australia), in which they recognized the main virulence factors hosted in regions with features linked to horizontal gene transfer. The *pld* gene (phospholipase D), the *FagABCD* operon, the *ciuABCD* operon, *glpT*, and the urea operon were identified.

Soares et al. (2012) [25] developed the PIPS (Pathogenicity Island Prediction) software package, which was the first package capable of using multiple features simultaneously for the integrated detection of pathogenicity islands [7]. The identification of the fourteen regions in the Mexican genomes was most similar to the results obtained by Soares et al. (2013) [6], who performed a pangenomic study with 15 strains of *C. pseudotuberculosis.* The PIPS software was used to predict PAI coordinates, identifying 16 PAI regions that were present in all isolates.

Among the main findings of the study by Soares et al. (2013) [6] was the identification of *pilus* genes (the groups *spaA* (*srtB-spaA-srtA-spaB-spaX-spaC*) and *spaD* (*srtC-spaD-spaY-spaE-spaF*)); however, despite search efforts, these genes were not identified in the Mexican genomes in our study. The absence of this group of important genes described in the genus could be due to various factors related to gene loss and acquisition within bacterial lineages. These events have previously been described in *Pseudomonas aeruginosa*, in which genes lost or acquired during the early years of infection were evaluated; gene loss is more frequent than gene acquisition [26]. The results were surprising because all lineages coincide in the loss or acquisition of the same genes, suggesting selection for the loss and acquisition of certain genes in the host environment. It is also important to mention that, despite the loss of genes observed in prokaryotes, the genome size appears to be relatively constant, a fact evidenced by the similarity of base pairs and genes identified between the reported strains and the recently sequenced Mexican genomes. Ely (2020) [27]. analyzed horizontal gene transfer events between two strains of *Caulobacter crescentus*, identifying the replacement of segments of the recipient genome rather than the simple addition of genetic material during horizontal gene transfer. The loss and acquisition of various genes in *C. pseudotuberculosis* should be further investigated through comparative genomics and horizontal gene transfer [27]. The search for virulence factors in *C. pseudotuberculosis* has been carried out in both the biovar *ovis* and biovar *equi* to determine the genetic material that allows the pathogen to develop infections in various hosts. Currently, virulence factors are being identified through databases such as the Virulence Factors Database (VFDB), which predicted a total of 418 virulence factors in a *Rhodococcus equi* genome (strain BJ13). It is worth noting that the most abundant group of genes identified were those belonging to iron uptake systems [28]. The enrichment of iron and metal-transport genes within the predicted PAIs is consistent with the critical role of metal acquisition in bacterial pathogenesis. During infection, the host deploys nutritional immunity, actively restricting the availability of essential metals such as manganese and zinc to invading pathogens [29]. The horizontal acquisition of additional metal uptake systems—including siderophore transporters (petrobactin), heme importers (*hmuU*), and ferric iron ABC transporters (*fag* operon) would provide a selective advantage for *C. pseudotuberculosis* in these metal-depleted intracellular niches. Abscess formation, the hallmark lesion of CLA, involves conserved pathogenic mechanisms shared with other abscess-forming bacteria; for example, mesenteric abscessation by *Pseudomonas aeruginosa* in equines demonstrates similar tissue tropism and inflammatory responses [30]. The concentration of these genes within PAIs suggests that their acquisition was a key event in the evolution of pathogenicity in this species, enabling colonization and persistence within host macrophages and lymph nodes.

For the first time in Mexican isolates, the genes comprising the Fag operon (*fagA, fagB, fagC*, and *fagD*) have been molecularly identified in complete sequences. This gene set has been used in the diagnosis of caseous lymphadenitis and in determining virulence in Mexican isolates [9]. The Fag operon sequences were identified immediately downstream of the *pld* gene. It should be noted that only the *FagC*, *FagD*, and *FagG* sequences were identified, while the *FagA* and *FagB* genes, although present in the genome, were not identified under characteristics linked to horizontal gene transfer, indicating greater genetic conservation in the genomic signature than in GIPSy, according to Baraúna et al. (2017) [31].

The *hmuT, hmuU*, and *hmuV* genes (ABC-type heme transporters) were identified in *C. diphtheriae* and *C. ulcerans*. It was verified in vitro that the *hmuT* gene binds to heme and hemoglobin-agarose, suggesting that it may participate as a heme captor in *C. diphtheriae*. The seventeen Mexican genomes harbored the *hmuU* gene, which encodes a permease protein of the heme transport system, in a pathogenicity island. The *hmuV* gene (ATP-binding protein that imports heme) was also identified in the genomes; however, it was not characterized within any PAIs [32]. The recognition of the island formed by the genes *yfmC* (Fe(3+) citrate-binding protein), *feuC* (iron absorption system permease protein), *fpuB* (petrobactin import system permease protein), and *fatE* (ATP-binding protein for petrobactin import) is one of the main findings of this research. Petrobactin is a siderophore involved in iron acquisition through binding to the membrane-associated substrate and other ABC-type transporters [33]. One of the main systems for iron absorption and bacterial survival in metal-restricted conditions is the production of high-affinity iron-chelating molecules called siderophores. Petrobactins are a key factor in the selection of new molecules considered for vaccine formulation. It has previously been postulated that a defective function of these molecules is associated with a reduction in bacterial pathogenicity [34].

The group of genes involved in iron acquisition has been a focus of study since the first reports of virulence genes being identified in complete genomes of the species *C. pseudotuberculosis*. The FRC41 strain was obtained from a sample of a twelve-year-old child from France diagnosed with necrotizing lymphadenitis; this strain is a reference for comparative studies between genomes obtained from various hosts. This study identified a group of genes also present in the genomes of *C. pseudotuberculosis* of Mexican origin obtained from sheep, goats, and cattle (*fagCBAD*, *ciuABCD*, and *HmuTUV* genes) [35].

An important finding of this analysis was the identification of the *phoB* gene (transcriptional regulatory protein) within the PAIs of Mexican isolates. Its involvement in the virulence of *Streptococcus agalactiae* was recently confirmed, where the deletion of *phoB* modified the expression of biofilm formation and virulence-related genes [36]. Another gene of interest is *artM* (ATP-binding protein for arginine transport), also identified in the genomes analyzed in this study, which was described in a group of genes involved in biofilm formation in *Haemophilus parasuis* [37]. This is interesting because there is scientific evidence of biofilm matrix formation by bacterial communities of *C. pseudotuberculosis*. A proteomic analysis identified 40 proteins involved in biofilm formation activity, among which those involved in cellular metabolism were the most abundant [38]. The identification of this gene may prompt new studies that result in the identification of targets for the treatment of CLA. In addition, two superoxide dismutases (SODs) encoded by the *sodA* and *sodC* genes were identified in the sequences of the seventeen Mexican genomes. Although they are not recognized in any PAI region, their involvement in the pathogenicity of *C. pseudotuberculosis* studied in mice has recently been demonstrated. The deletion of *sodA* and *sodC* decreased bacterial loads and histopathological lesions in infected organs. Specifically, the deletion of *sodC* reduced the ability to form biofilms, the survival of the pathogen within macrophages, and the ability to resist oxidative stress [39].

The identification of PAI-associated virulence genes has direct translational implications. The *pld* gene, encoding phospholipase D, remains the most validated diagnostic and vaccine target for CLA [8]. Our identification of petrobactin transporter genes (*fpuB*, *fpuC*) and the fag operon within PAIs provides additional candidate antigens for subunit vaccine formulation, given that disruption of siderophore systems has been associated with reduced pathogenicity [34]. The *phoB* and *artM* genes, implicated in biofilm formation in related pathogens [36,37], represent potential targets for anti-biofilm therapeutic strategies. Furthermore, the positively selected genes *radA* and *rpiB* could serve as novel drug targets, as demonstrated for *RpiB* in *Leishmania infantum* [23,24]. Recent advances in antigen-based serodiagnosis for CLA [40] and the evaluation of non-antibiotic control strategies such as medicinal plant extracts against *C. pseudotuberculosis* [41] further support the translational relevance of identifying and characterizing virulence determinants in regional strain variants.

Pathogenicity island prediction analyses must be updated as the number of available sequenced genomes grows. These studies will provide information about adaptation and new host–pathogen interactions that may arise as horizontal gene transfer occurs [42]. Pathogenicity island studies have had a significant impact on important pathogenic species such as *Salmonella enterica* and *E. coli*, pathogens that are currently developing multidrug-resistant and virulent clones, creating an urgent need to understand the processes driving bacterial evolution and the acquisition of virulence and antibiotic resistance genes [43]. Additionally, recent work by Naeem et al. (2025) [41] demonstrated the antimicrobial potential of medicinal plant extracts against *C. pseudotuberculosis* in vitro, underscoring the need for alternative control strategies informed by virulence gene characterization [41]. The broader One Health implications of virulence factor dissemination through horizontal gene transfer in veterinary pathogens have been highlighted by studies documenting antimicrobial resistance and virulence determinants in foodborne bacteria from animal production systems [44].

In the present study, in addition to predicting pathogenicity islands and their harbored genes, we analyzed the sequence similarity of these regions between Mexican isolates and other pathogenic strains of the genus *Corynebacterium*, which resulted in high percentages of sequence similarity, suggesting the conservation of these regions, described as variable and unstable, at least in strains originating from the same geographic site.

### 4.3. Limitations

This study has several limitations that should be acknowledged. First, all genome assemblies are draft quality (contigs from short-read Illumina sequencing), which may affect the precise delineation of PAI boundaries, flanking tRNA identification, and the detection of genes located at contig edges. Second, PAI prediction relied solely on GIPSy; complementary tools (e.g., IslandViewer, Alien_Hunter) could strengthen predictions through cross-validation. Third, the virulence factors and positively selected genes identified herein were characterized computationally; experimental validation through gene knockout, complementation, and animal infection models is essential to confirm their functional roles. Fourth, the absence of accession numbers deposited at the time of submission limits immediate reproducibility; although the genome sequences have a preregistration in NCBI. Fifth, the sample originates from a single geographic region in Mexico, which may not capture the full genetic diversity of *C. pseudotuberculosis* biovar *ovis* circulating in the country. Sixth point, the positive selection analysis was computational and based on codon-model inference using POTION, a genome-scale pipeline designed to detect candidate genes under positive Darwinian selection while reducing false positives through filtering and multiple-testing correction. Therefore, the five positively selected coding sequences represent candidate adaptive loci and require experimental validation. Another key limitation of this study is the lack of experimental validation of the virulence factors identified in the predicted PAIs. Future studies should employ gene deletion, complementation, and infection models to confirm their role in pathogenesis. Finally, the wgMLST dendrogram represents genomic relatedness based on allelic-profile distances and should not be interpreted as a deep phylogenomic reconstruction.

## 5. Conclusions

In this study, we sequenced and analyzed seventeen complete genomes of *Corynebacterium pseudotuberculosis* biovar *ovis* isolated in Mexico. Comparative genomics revealed high intra-biovar sequence conservation. This finding confirms the pathogen’s clonal behavior. Phylogenetic profiling grouped two Mexican isolates with strains from different host species. We mapped fourteen putative pathogenicity islands (PAIs) that harbor critical virulence factors. These factors are predominantly genes involved in metal ion acquisition and transport, such as the *Fag* operon and petrobactin transporters. Additionally, positive selection analysis identified five coding sequences, including *radA* and *rpiB*, that are undergoing adaptive evolutionary changes. These findings elucidate the pathogenic mechanisms and genomic plasticity of Mexican *C. pseudotuberculosis* strains. They also highlight novel genetic targets for vaccine and therapeutic development against caseous lymphadenitis.

## 6. Patents

The genomic sequences reported in this study have been deposited with the National Center for Biotechnology Information (NCBI) under BioProject accession number PRJNA1468863 (submission ID: SUB16201636).

## Figures and Tables

**Table 1:** Clinical characteristics of *Corynebacterium pseudotuberculosis* biovar *ovis* isolates obtained from abscess lesions in different animal species.

Identification of the Isolate	Abscess Size		Type of Exudate				Species			Sex		Obtaining Site				Municipality				State	
	<5 cm	>5.1 cm	Serous	Seropurulent	Purulent	Caseous	Sheep	Goat	Cow	Female	Male	Head	Neck	Groin	Liver	Zapotlanejo	Zapopan	Tepatitlan	Toluca	Jalisco	State of Mexico
9-19	1		1				1			1		1				1				1	
1-6 2L-J	1			1			1			1		1				1				1	
8-19 2L-J	1			1			1			1		1				1				1	
585790	1			1			1			1		1						1		1	
H14X		1			1		1			1		1						1		1	
(030) 8150		1			1		1			1			1				1			1	
(039) 978	1				1		1			1			1				1			1	
(047) 104W	1				1		1			1			1				1			1	
(033B) 635816	1		1				1			1			1			1				1	
3612PK		1	1				1			1			1					1		1	
9575R	1		1				1			1			1			1				1	
(005) B48W2009	1					1	1			1			1			1				1	
728930	1			1			1			1			1					1		1	
728905	1			1			1				1		1					1		1	
59 (1-6)	1			1				1		1				1		1				1	
(2-4) 668478	1			1				1		1		1				1				1	
CP VACA	1				1				1						1				1		1
Total	14	3	4	7	5	1	14	2	1	15	2	6	9	1	1	8	3	5	1	16	1
%	82.3	17.6	23.5	41.1	29.4	5.8	82.3	11.7	5.8	88.2	11.7	35.2	52.9	5.8	5.8	47.05	17.6	29.4	5.8	94.1	5.8

Data are presented as the number of *Corynebacterium pseudotuberculosis* biovar *ovis* isolates obtained from abscess lesions in different animal species according to abscess size, type of exudate, host species, sex, anatomical site of sample collection, municipality, and state of origin. Abscess size was classified as <5 cm or >5.1 cm. Exudate types included serous, seropurulent, purulent, and caseous. The anatomical sites of isolation included head, neck, groin, and liver. Percentages were calculated based on the total number of isolates analyzed (n = 17). The predominance of female animals in our sample (88.23%) reflects the composition of the sampled flocks and does not permit statistical comparison of genomic features between sexes.

**Table 2:** Genomic characteristics of *Corynebacterium pseudotuberculosis* biovar *ovis* obtained from abscessed lesions in sheep, goats and cattle, using the Illumina technique.

Isolated	Genomic Size (bp)	Genes	G + C Content %
(9-19)	2,337,363	2135	52.18
56 (1-6)	2,332,007	2129	52.18
1-6 2L-J	2,337,302	2132	52.18
(2-4) 668478	2,331,651	2134	52.18
(005) B48W2009	2,337,498	2135	52.18
8-19 2L-J	2,331,551	2130	52.18
(030) 8150	2,337,345	2136	52.18
039 978	2,331,722	2133	52.18
(047) 104W	2,331,574	2135	52.18
H14X	2,337,569	2135	52.18
(033B) 635816	2,331,531	2131	52.18
3612PK	2,337,413	2132	52.18
9575R	2,337,612	2132	52.18
585790	2,337,637	2138	52.18
728905	2,331,674	2132	52.18
728930	2,331,674	2132	52.18
CP VACA	2,337,436	2135	52.18

**Table 3:** Genes and proteins harbored in the putative pathogenicity islands of *Corynebacterium pseudotuberculosis* of Mexican origin.

Gene	Protein
Genes involved in the acquisition of metals and other nutrients	
*nikA*	Nickel-binding protein NikA
*copa*	Copper-exporting P-type ATPase
*ctpC*	Manganese-exporting P-type ATPase
*nikE*	Nickel import from the ATP-binding protein NikE
*btuD*	Vitamin B12 imports the ATP-binding protein BtuD
*nikB*	Nickel transport system permease protein NikB
*btuC*	Vitamin B12 import system protein permease BtuC
*troA*	Periplasmic zinc-binding protein TroA
*scaC*	ATP-binding protein Manganese import
*mntD*	Membrane protein of the manganese transport system MntD
*btuF*	Vitamin B12 binding protein
Genes involved in iron uptake systems	
*hmuU*	Hemin transport system permease protein HmuU
*yfmC*	Fe(3+) citrate-binding protein YfmC
*feuC*	Iron absorption system permease protein FeuC
*fpuB*	Petrobactin protein permease FpuB import system
*fpuC*	Petrobactin imports ATP-binding protein FpuC
*fecE*	Fe(3+) dicitrate transport ATP-binding protein FecE
*fagC*	Ferric enterobactin transport ATP-binding protein FepC
*fagG*	Ferric enterobactin transport system permease protein FepG
*fagD*	Ferric enterobactin transport system permease protein FepD
Toxin genes	
*pld*	Phospholipase D
Transposase-type genes	
*IS256*	IS256 ISGsi1 family transposase
Urea operon	
*ureD*	Accessory protein urease UreD
*ureG*	Accessory protein urease UreG
*ureF*	Accessory protein urease UreF
*ureE*	Accessory protein urease UreE
*ureC*	Urease alpha subunit
*ureB*	Urease beta subunit
*urea*	Urease gamma subunit
Genes involved in antimicrobial resistance	
*mdtH*	Multidrug resistance protein MdtH
*lnrL*	ATP-binding protein LnrL/Linearmycin resistance
*bcrA*	Bacitracin transport ATP-binding protein BcrA
Genes involved in bacterial metabolism	
*oppF*	Oligopeptide transport ATP-binding protein OppF
*artM*	Arginine transport ATP-binding protein ArtM
*appA*	AppA oligopeptide binding protein
*lysX*	Biosynthesis of lysylphosphatidylglycerol bifunctional protein
*deoR*	Deoxyribonucleoside regulator
*deoD*	Purine nucleoside phosphorylase type DeoD
*deoC*	Deoxyribose-phosphate aldolase
*lysC*	Aspartokinase
*phoB*	Phosphate regulon transcriptional regulatory protein PhoB
*kdpD*	KdpD sensor protein
*degU*	DegU transcriptional regulatory protein
*que*	7-carboxy-7-deazaguanine synthase
*dapC*	Putative N-succinyldiaminopimelate aminotransferase DapC
*lysX*	Biosynthesis of lysylphosphatidylglycerol bifunctional protein
*rkpK*	UDP-glucose 6-dehydrogenase
*dcd*	dCTP deaminase, forming dUMP
*gabD*	Succinate semialdehyde dehydrogenase [NADP (+)] 1
*glpQ*	Glycerophosphodiester phosphodiesterase
*glpT*	Glycerol-3-phosphate transporter

Genes were grouped according to their predicted biological functions, including metal and nutrient acquisition, iron uptake systems, toxins, transposases urea operon, antimicrobial resistance, and bacterial metabolism.

## Data Availability

The original contributions presented in this study are included in the article. Further inquiries can be directed to the corresponding author.

## Supplementary Materials

The following supporting information can be downloaded at: https://www.mdpi.com/article/10.3390/cimb48060598/s1.

## Abbreviations

The following abbreviations are used in this manuscript:

CLA	Caseous lymphadenitis
PAIs	Pathogenicity islands
THG	Horizontal gene transfer
