# Supplementary material for: Genomic Characterization and Pathogenicity Island Analysis of 17 Mexican Isolates of Corynebacterium pseudotuberculosis biovar ovis
Source: Curr Issues Mol Biol. 2026 Jun 5;48(6):598. doi: 10.3390/cimb48060598 (PMC13298101; doi:10.3390/cimb48060598)
Supplement: Supplementary file 1 [file cimb-48-00598-s001.zip › cimb-4306497-supplementary.pdf]

Supplementary Materials

Table S1. Information on the strains taken from GenBank used

**Cepa**	**Biovar**	**Host**	**País de origen**	**Descripción clínica**	**Identificación GenBank**
1002	*ovis*	Cabra	Brasil	Absceso LAC	CP001809
C231	*ovis*	Oveja	Australia	Absceso LAC	CP001829
42/02-A	*ovis*	Oveja	Australia	Absceso LAC	CP003062
PAT10	*ovis*	Oveja	Argentina	Absceso	CP002924
3/99-5	*ovis*	Oveja	Escocia	LAC	CP003452
267	*ovis*	Llama	Estados Unidos	Absceso LAC	CP003407
P54B96	*ovis*	Antílope	África	Absceso LAC	CP003385
I19	*ovis*	Vaca	Israel	Absceso Mastitis Bovina	CP002251
FRC41	*ovis*	Humano	Francia	Linfadenitis necrosante	CP002097
CIP52.97	*equi*	Caballo	Kenia	Linfangitis ulcerativa	CP003061
316	*equi*	Caballo	Estados Unidos	Absceso	CP003077
258	*equi*	Caballo	Bélgica	Linfangitis ulcerativa	CP003540
1/06-A	*equi*	Caballo	Estados Unidos	Absceso	CP003082
Cp162	*equi*	Camello	Reino Unido	Absceso	CP003652
31	*equi*	Búfalo	Egipto	Absceso	CP003421

Corynebacterium glutamicum ATCC13032.
Corynebacterium ulcerans NCTC13718.
Corynebacterium diphtheriae NCTC131529.

Metrics of the seventeen sequenced Mexican isolates.

Isolate 9-19: Average depth = 11.4958X; Standard variation = 4.4546; Readings after quality filter 116915; reads mapped: 116774. Isolate (59) 1-6 : Average depth = 13.1509X; Standard variation = 4.89966; Readings after quality filter 134926; reads mapped: 134751. Isolate 1-6 2L-J: Average depth = 15.7828X; Standard variation = 5.45794; Readings after quality filter 161117; reads mapped: 160935. Isolate (2-4)668478: Average depth = 14.5381X; Standard variation = 5.34844; Readings after quality filter 149113; reads mapped: 148914. Isolate 005: Average depth = 17.1068X; Standard variation = 5.81873; Readings after quality filter 175815; reads mapped: 175621. Isolate 8-19-2L-J: Average depth = 17.3096x; Standard variation = 5.9157; Readings after quality filter 176548; reads mapped: 176330. Isolate 030: Average depth = 12.8038X; Standard variation = 4.86439; Readings after quality filter 130383; reads mapped: 130238. Isolate 039: Average depth = 21.2249X; Standard variation = 6.73676; Readings after quality filter 215203; reads mapped: 215041. Isolate 047: Average depth = 17.2082X; Standard variation = 6.0631; Readings after quality filter 182804; reads mapped: 182624. Isolate 1414X: Average depth = 10.6003X; Standard variation = 4.31267; Readings after quality filter 107264; reads mapped: 107160. Isolate 03386: Average depth = 13.8519X; Standard variation = 5.14264; Readings after quality filter 141469; reads mapped: 141333. Isolate 3612PK: Average depth = 13.8107X; Standard variation = 5.04216; Readings after quality filter 140926; reads mapped: 140759. Isolate 9575R: Average depth = 13.9957X; Standard variation = 5.14221; Readings after quality filter 144671; reads mapped: 144417. Isolate 58579: Average depth = 12.8769X; Standard variation = 4.88689; Readings after quality filter 129474; reads mapped: 129341. Isolate 728905: Average depth = 13.2135X; Standard variation = 4.72891; Readings after quality filter 132239; reads mapped: 132067. Isolate 728930: Average depth = 12.9806x; Standard variation = 4.87056; Readings after quality filter 131371; reads mapped: 131169. Isolate CPVACA: Average depth = 13.8914X; Standard variation = 5.27178; Readings after quality filter 148374; reads mapped:148231.
